# Lifestyle including dietary habits and changes in coronary artery calcium score: a retrospective cohort study

**DOI:** 10.1186/s40885-016-0038-9

**Published:** 2016-01-28

**Authors:** EunSun Cheong, Jong-Young Lee, Sung Ho Lee, Jin-Ho Kang, Bum-Soo Kim, Byung Jin Kim, Ki-Chul Sung

**Affiliations:** Division of Cardiology, Department of Medicine, Kangbuk Samsung Hospital, Sungkyunkwan University, School of Medicine, #108, Pyung Dong, Jongro-Ku, Seoul, 110-746 Republic of Korea

**Keywords:** Dietary habits, Lifestyle, Coronary artery calcium score, Macronutrient, Physical activity, Depression

## Abstract

**Background:**

General diet and lifestyle are considered to have an effect on levels of atherosclerosis but previous studies have shown inconsistent results. The aim of this study was to investigate whether macronutrient intake, physical activity and depressive symptoms are associated with progression of preclinical atherosclerosis in healthy Korean adults.

**Methods:**

A total of 2623 individuals from Kangbuk Samsung Hospital Health Screening Center in South Korea were enrolled between 2010 and 2012 and had follow-up at 2013. Every participant received a non-enhanced coronary computed tomography (CT) and completed questionnaires for food intake frequency, depression and physical activity levels. The study population was divided into two groups according to CAC progression, namely CAC group (CAC score >0) or non-CAC group (CAC score ≤ 0), and were compared according to macronutrient intake, degree of depressive symptoms and physical activity.

**Results:**

A total of 2175 subjects were eligible for the analysis and among them, 592 subjects had progression of CAC. Total energy, carbohydrate and fat intake showed significant differences between the two groups (p-values of 0.01, 0.021 and 0.016 respectively). However, levels of protein intake did not vary for the two groups (*p* = 0.286). Depressive symptoms and extent of physical activity were similar between the two groups. Multivariate analysis was conducted with adjustment for possible confounding factors. The hazard ratios for CAC progression were not different according to macronutrient intake, degree of depressive symptoms and physical activity.

**Conclusion:**

In this large relatively healthy population based observational study, CAC progression showed no significant association with total energy intake, proportion of macronutrient intake, depressive symptom and physical activity.

**Electronic supplementary material:**

The online version of this article (doi:10.1186/s40885-016-0038-9) contains supplementary material, which is available to authorized users.

## Background

Coronary artery calcification (CAC) is regarded as important pathognomonic sign of coronary atherosclerosis [1]. The presence of subintimal coronary calcification results from a healing process after rupture of unstable lesions from atheroma [2, 3] and measurement of CAC by multidetector computed tomography (MDCT) is widely used as a non-invasive tool for assessing atherosclerotic plaque burden in the coronary arteries.

Although age, sex, hypertension, dyslipidemia, diabetes, glucose intolerance, smoking, obesity are all known risk factors for developing CAC, they are inconsistent predictors [4–7]. This suggests that other unrecognized risk factors may be present for developing CAC. It is plausible that dietary factors increase the risk of CAC and previous findings have shown that cardiovascular calcification is related to intake of starch or sugar in animals [8] and similarly, a high glucose medium enhances calcification of human vascular smooth muscle cells and increases expression of markers of bone formation [9]. Also from a recent report citing Cochrane database of trials, excessive fat intake may have an adverse effect on coronary vascular disease (CVD) [10].

Apart from dietary intake, other lifestyle habits may be associated with CAC. A number of studies have reported an association with excessive sleep duration [11, 12] and physical activity with carotid or coronary atherosclerosis [13–15]. Also, depression may be a possible risk factor [16, 17]. It should be noted that these studies also show some inconsistent results.

Previously, we found that composition of dietary macronutrient intake was not associated with a prevalence of coronary artery calcification in healthy Korean adults [18]. The conclusion, however, may not have been definitive as the study was from a cross-sectional analysis. In this study, we hypothesized that the daily intake of macronutrients such as carbohydrate, protein and fat and physical activity or depressive symptoms may show a correlation with progression of CAC in healthy population at low risk of CVD.

## Methods

### Study population

The participants consisted of individuals older than 18 years who underwent a baseline comprehensive health examination at Kangbuk Samsung Hospital Health Screening Center in Seoul and Suwon, South Korea, between 2010 and 2012 and had follow up at 2013. A total of 2623 individuals were identified to have received non-enhanced coronary computed tomography, completed the food frequency questionnaire (FFQ) in addition to questionnaires for depressive symptoms and physical activity at baseline and follow up. The following participants were excluded from analysis: 379 subjects with missing data (on smoking, exercise, and alcohol intake), 44 subjects with a history of cancer, and 52 subjects with a history of cardiovascular disease. As some individuals met more than one criterion for exclusion, the total number of eligible subjects for the study was 2175. Among these subjects we could obtain the answer for questionnaires; 1099 subjects for total energy intake, 917 subjects for composition of macronutrients, 907 subjects for sleep duration and 864 subjects for depression. In Korea, the Industrial Safety and Health Law requires employees to participate in annual or biannual health examinations. Approximately 60 % of the participants were employees of various companies and local governmental organizations.

This study was approved by the Institutional Review Board (IRB) of Kangbuk Samsung Hospital and informed consent requirement was waived as all personal identifiable information was removed prior to accession.

### Measurements

#### Anthropometric measurements and general characteristics

Body weight and height of subjects were measured to the nearest 0.1 kg and 0.1 cm, respectively. Body mass index (BMI) was calculated as weight in kilograms divided by the square of the height in meters. Obesity was defined as BMI ≥ 25 kg/m^2^. Data on past medical history, medication use, and health-related behaviors were obtained by a self-administered questionnaire. Questionnaires were used to evaluate education level, smoking status (current or non-current), alcohol consumption (frequency per week, amount) and sleep duration (hours per day). Depression was evaluated by CES-DK score [19] and food frequency data was calculated by CAN-pro 4.0 (Korean Nutrition Society 2010).

Alcohol intake was examined as unit per day of alcohol consumption. Smokers were divided into two groups: current smoker and non-current smoker. Physical activity was evaluated using the Korean version of the International Physical Activity Questionnaire (IPAQ) short form [20, 21]. The number of days per week and time spent walking per day, as well as moderate and vigorous activities were recorded. The collected data were converted to metabolic equivalent scores (METS) for each type of activity. By multiplying the time engaged in the activity in a week with consideration to the number of METs, metabolic equivalent task minutes per week, MET-min/week, were calculated according to the IPAQ scoring protocol [22]. Blood pressure was measured with electronic sphygmomanometer in the seated position with more than 5 min of resting prior to the measuring.

#### Coronary Artery Calcification (CAC) measurement

Coronary artery calcification (CAC) was detected by a LightSpeed VCT XTe-64 slice MDCT scanner (GE Healthcare, Tokyo, Japan) with the same standard scanning protocol using 2.5-mm thickness, 400 ms rotation time, 120 kV tube voltage, and 124 mAs (310 mA * 0.4 s) tube current under ECG-gated dose modulation. Coronary artery calcification was defined as more than three contiguous pixels above a CT density of 130 Hounsfield Units. The total CAC score was calculated by Agatston's method [23]. Subjects were classified into two subgroups according to CAC score: CAC group (CAC score >0) or non-CAC group (CAC score ≤ 0) by referring to previous studies.

#### Biochemical marker

Blood samples were taken from the antecubital vein, collected in serum-separating tube (SST) after at least 10 h of fasting. Serum levels of total cholesterol and triglyceride were determined using an enzymatic colorimetric assay; low-density lipoprotein cholesterol (LDL-C) and high-density lipoprotein cholesterol (HDL-C) levels were directly measured using a homogeneous enzymatic colorimetric assay. Serum fasting glucose level was measured using the hexokinase method. Fasting serum glucose, total cholesterol, LDL-C, HDL-C, triglyceride (TG), alanine aminotransferase (ALT), gamma-glutamyl transferase (GGT), were measured using Bayer Reagent Packs in an automated chemistry analyzer (Advia 1650 Auto analyzer; Bayer Diagnostics, Leverkusen, Germany). High sensitivity C-reactive protein (hsCRP) was analyzed by particle-enhanced immunonephelometry with the BNII System (Dade Behring, Marburg, Germany). All hematologic measurements were analyzed in one laboratory with the same machines by trained staff using the same methodology throughout. The Korean Society of Laboratory Medicine (KSLM) biannually certified the Laboratory Medicine Department at Kangbuk Samsung Hospital in Seoul, Korea for the Korean Association of Quality Assurance for Clinical Laboratories (KAQACL) and the CAP (Collage of American Pathologists) Proficiency Testing designations.

#### Nutrient intake measurements

Self-administered food frequency questionnaire (FFQ) was used to obtain nutrient intake data which was designed and validated for use in Korea. Food frequency was estimated by 9 scales (never, 1 time/month, 2 – 3 times/month, 1 – 2 times/week, 3 – 4 times/week, 5 – 6 times/week, 1 time/day, 2 times/day and 3 times/day) and portion size was estimated by three scales (half dish, one dish, one and a half dish) for consumption of 103 food items over the past year. Nutrient intake data include total energy (kcal), carbohydrate (g), protein (g) and fat (g). The Food Composition Table, a nutrient database produced by the Korean Nutrition Society to convert food intake into nutrients, was used to perform nutrient analysis [24]. The contribution of each macronutrient to energy was calculated as the ratio of energy from each macronutrient to total energy: percentage of carbohydrate from total energy intake (%), percentage of fat from total energy intake (%) and percentage of protein from total energy intake (%). Food frequency data was calculated by CAN-pro 4.0 (Korean Nutrition Society 2010). Subjects were categorized into four groups according to each macronutrient intake (Q1 ~ Q4).

### Statistical analyses

Normally distributed variables are presented as the mean ± SD and skewed variables are presented as the median (interquartile range). Continuous variables were compared using independent *t*-test between CAC change ≤ 0 group and CAC change > 0 group (CAC progression). Categorical variables were expressed as number and percentages then compared between groups using the χ 2 -test. Multiple logistic regression analysis were used to determine Hazard Ratios (HRs) for CAC progression with 95 % confidence intervals (CIs) for quartile groups of each total energy intake, macronutrient intake, physical activity and depression using the lowest quartile group as the reference. To evaluate the significance, two models were constructed. Model 1 was adjusted for age and sex and Model 2 was adjusted for Model 1 and smoking, physical activity, alcohol intake, glucose, triglyceride, HDL-cholesterol, LDL-cholesterol, and blood pressure. P values <0.05 were considered statistically significant. The STATA 11.2 software package was used for statistical analysis.

## Results

**Fig. 1:**
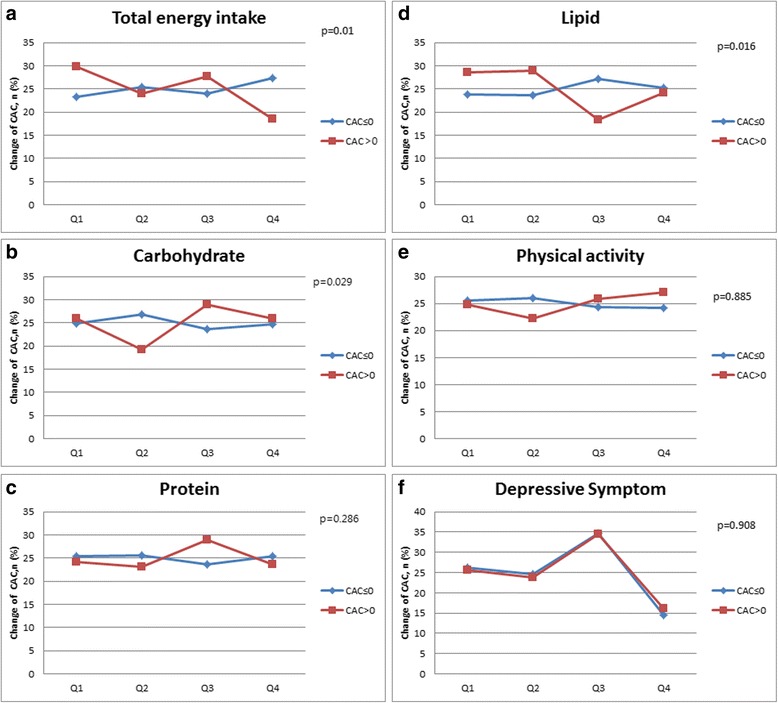
Associations of total energy, macronutrients, physical activity and depression according to coronary artery calcium change. **a** Correlation between CAC change and total E intake. **b** Correlation between CAC change and carbohydrate intake. **c** Correlation between CAC change and protein intake. **d** Correlation between CAC change and lipid intake. **e** Correlation between CAC change and physical activity. **f** Correlation between CAC change and depression

A total of 2175 participants were divided into two groups according to CAC change and 592 subjects showed CAC progression with CAC change > 0. Mean follow-up duration was 2.16 ± 0.15 years. Baseline characteristics of these groups are illustrated in Table 1. Majority of the participants were male (97 %) and mean age of CAC change ≤ 0 group and CAC change > 0 group were 41.6 ± 5.6 years and 44.8 ± 5.5 years, respectively. CAC progression group were older, with higher BMI, level of obesity, diabetes, hypertension, alcohol intake and adverse lipid profiles (all *p* < 0.001). However current smoking status did not differ between the groups. Daily total energy intake for CAC change ≤ 0 group and CAC change > 0 group were 1512.8 and 1473.7 kcal respectively (*p* < 0.005). However the proportion of carbohydrate, protein and fat intake were not different between the two groups. Mean physical activity of the CAC change ≤ 0 group and CAC change > 0 group were 887.5 and 1045.5 METS per day, respectively (*p* = 0.02) and depression score (CES-D score) were similar with 4 and 5 for each group (*p* = 0.307). We compared the two groups according to quartile of total daily energy intake, each macronutrient intake and physical activity and depressive symptoms (Fig. 1). Total energy, carbohydrate and fat intake showed significant difference between the two groups with p value of 0.01, 0.029 and 0.016 respectively. However, proportion of protein intake did not vary with p value of 0.286. Depressive symptoms and extent of physical activity were similar between the groups. Additional analysis of sleep duration and alcohol intake showed significant difference for alcohol intake (*p* = 0.001) but not for sleep.

**Table 1:** Baseline characteristics of study participants by CAC change

	CAC change		p-value
	CAC change ≤ 0	CAC change > 0	
Number	1,583	592	
Age (years)^a^	41.6(5.6)	44.8(5.5)	<0.001
Male (%)	93.6	99.2	<0.001
BMI(kg/m^2^)	24.9(3.0)	25.8(3.0)	<0.001
Obesity (%)	45.4	56.9	<0.001
Current smoker (%)	33.2	35.6	0.288
Alcohol intake (%)^b^	32.3	40.7	<0.001
High education level (%)^c^	85.5	87.0	0.404
Diabetes (%)	6.3	15.0	<0.001
Hypertension (%)	20.3	33.6	<0.001
Systolic BP (mmHg) ^a^	118.5(12.2)	121.3(11.9)	<0.001
Diastolic BP (mmHg)^a^	75.9(9.4)	78.1(9.5)	<0.001
Glucose (mg/dL)^a^	98.6(15.4)	103.9(21.9)	<0.001
Total cholesterol (mg/dL)^a^	206.8(36.4)	218.6(38.0)	<0.001
LDL-C (mg/dL)^a^	130.0(32.7)	141.1(34.2)	<0.001
HDL-C (mg/dL)^a^	51.7(12.4)	49.3(11.1)	<0.001
Triglycerides (mg/dL)^d^	130(89–190)	153(110.5–215)	<0.001
ALT (U/l)^d^	25(18–37)	28(20–40)	<0.001
GTP (U/l)^d^	34(22–54)	40(27–65)	<0.001
C-reactive protein (mg/L) ^d^	0.06(0.03–0.11)	0.07(0.04–0.13)	0.022
Total energy	1530.9(1055.0–1933.0)	1483.5(659.4.2–1788.9)	0.001
Carbohydrate %	74.7(69.0–80.0)	76.0(68.6–80.3)	0.497
Protein%	13.8(12.5–15.2)	13.9(12.6–15.0)	0.966
Fat%	16.3(12.7–20.3)	14.9(12.3–20.2)	0.244
Physical activity (MET-min/week)	887.5 (396–1836)	1045.5 (438–1977)	0.800
Depression (CES-D score)	4 (1–9)	5 (1–9)	0.283

Data are ^a^means (standard deviation), ^d^medians (interquartile range), or percentages

Abbreviations: ALT, alanine aminotransferase; BMI, body mass index; BP, blood pressure; LDL-C, low-density lipoprotein-cholesterol; HDL-C, high-density lipoprotein-cholesterol; GTP, gamma glutamyl transferase

^b^ ≥20 g/day; ^c^ ≥ College graduate

As a few macronutrient and alcohol intake showed significant differences, multivariate analysis was conducted in CAC change > 0 group according to quartiles of total energy, macronutrient intake, physical activity and depressive symptom to confirm relationship between those variables mentioned above and CAC progression. Model 1 and model 2 were suggested with adjustment of possible confounding factors (age, sex, smoking, physical activity, alcohol intake, glucose, triglyceride, HDL-cholesterol, LDL-cholesterol, and blood pressure) (Tables 2, 3). However, after adjustment of possible confounding factors, the HRs for CAC change > 0 were not significant in all categories with insignificant p values for trends in both models. We conducted a sub-analysis only including those with more than two years of follow up however it did not alter the result (Additional file 1).

**Table 2:** Multivariate analysis for coronary artery calcification score according to quartile of macronutrients intake

	Q1	Q2	Q3	Q4	P for trend
Total energy					
N (total /CAC change > 0)	275/87	275/70	275/81	274/54	
Model 1^a^	1.00	0.73(0.50–1.08)	1.01(0.69–1.48)	0.71(0.47–1.08)	0.317
Model 2^b^	1.00	1.09(0.61–1.94)	1.25(0.70–2.22)	1.02(0.56–1.87)	0.826
Carbohydrate %					
N (total /CAC change > 0)	230/59	229/44	229/66	229/59	
Model 1^a^	1.00	0.67(0.42–1.06)	1.12(0.72–1.73)	0.79(0.50–1.23)	0.764
Model 2^b^	1.00	0.63(0.34–1.18)	1.58(0.86–2.91)	0.90(0.48–1.68)	0.581
Protein%					
N (total /CAC change > 0)	230/55	229/53	229/66	229/54	
Model 1^a^	1.00	0.99(0.63–1.56)	1.42(0.92–2.21)	0.89(0.57–1.41)	0.951
Model 2^b^	1.00	1.09(0.58–2.07)	1.18(0.63–2.21)	0.68(0.36–1.27)	0.252
Fat%					
N (total /CAC change > 0)	230/55	229/53	229/66	229/54	
Model 1^a^	1.00	1.32(0.85–2.03)	0.75(0.47–1.20)	1.07(0.69–1.68)	0.658
Model 2^b^	1.00	1.88(1.01–3.51)	0.58(0.30–1.13)	0.99(0.52–1.86)	0.238

Data are adjusted hazard ratios with 95 % CI

^a^ Model 1: Adjusted for age and sex. ^b^ Model 2: Model 1+ smoking, physical activity, alcohol intake, glucose, triglyceride, HDL-cholesterol, LDL-cholesterol, waist circumference, blood pressure

**Table 3:** Multivariate analysis for coronary artery calcification score according to physical activity and depression

	Q1	Q2	Q3	Q4	P for trend
Physical activity					
N (total /CAC change > 0)	270/70	266/63	263/73	265/76	
Model 1^a^	1.00	0.90(0.60–1.36)	1.00(0.67–1.50)	1.01(0.68–1.52)	0.833
Model 2^b^	1.00	0.99(0.56–1.74)	1.12(0.63–1.98)	1.04(0.58–1.85)	0.894
Depression					
N (total /CAC change > 0)	225/64	211/59	299/86	129/40	
Model 1^a^	1.00	0.92(0.60–1.43)	0.90(0.60–1.35)	1.09(0.66–1.79)	0.927
Model 2^b^	1.00	1.57(0.83–2.99)	0.93(0.51–1.71)	1.66(0.83–3.33)	0.478

Data are adjusted hazard ratios with 95 % CI

^a^ Model 1: Adjusted for age and sex. ^b^ Model 2: Model 1+ smoking, physical activity, alcohol intake, glucose, triglyceride, HDL-cholesterol, LDL-cholesterol, blood pressure

## Discussion

In this relatively healthy cohort of Korean adults, total energy intake, proportion of carbohydrate, protein, fat, physical activity and depression were not associated with progression of CAC after adjustment of possible confounding factors.

A few studies have investigated the relationship between macronutrient intake and CAC or other markers of subclinical atherosclerosis [8, 18, 25]. Recent report from Cochrane database showed excessive fat intake has an adverse effect on coronary vascular disease (CVD) [10]. On the other hand, a large cross-sectional population-based study reported that healthy dietary pattern was not related to CAC with carotid artery IMT after adjustment for various confounders [25]. Recently, we also suggested that the prevalence of CAC may not be associated with composition of macronutrient intake [18]. Although previous studies have studied the relationship between macronutrient intake and CAC, to the best of our knowledge, our study is the first to analyze the relationship between macronutrient intake and progression of CAC. An important finding in our study is that high carbohydrate and fat diet did not affect CAC progression. This result is the same as our previous study [18].

There are several published data on a potential benefit of physical activity on atherosclerosis [13, 14, 26]. A population-based study found that vigorous physical activity had an inverse association with incident CAC [13] and a prospective study reported that maintaining regular high physical activity level might protect against atherosclerosis [14].

From a study of a small number of spinal cord injury (SCI) cases, able-bodied SCI patients had improved common carotid artery intima-media thickness (IMT) compared with sedentary SCI [26]. Several studies have also revealed a positive effect of physical activity on reducing level of many atherosclerotic factors such as CRP and cytokines [27]. However, our study failed to show a significant association between physical activity and progression of atherosclerosis. Although the overall association between CAC progression and physical activity was found to be insignificant, there were a positive trend of higher level of physical activity in CAC change > 0 group whereas physical activity tended to be lower in CAC change ≤ 0 group. One possible explanation is that as the BMI of CAC change > 0 group was higher than that of the other group, the individuals may have tried to be more active and lose more weight.

Cardiovascular disease (CVD) and comorbid depression seem to have a bidirectional relationship. Previous etiological and prognostic studies have suggested that depression may worsen the severity of CVD and also contribute to the onset of the disease, thus impacting CVD in multiple ways [16, 17]. The interaction of CVD and depression may involve experience of chronic stress triggering neurohormonal activation, autonomic neurocardiac dysfunction with the ensuing cytokine cascades leading to chronic inflammation [28]. Despite the previous studies, our result with depressive symptoms failed to show a significant association, which may be explained as follows. CES-D scale, which is designed to measure depressive symptoms in general population, has more than 16 points considered to be depressive in Western population [29]. However the cutoff for Koreans is considered to be 21 points and higher than Western population [19] and may be due to the sociocultural differences. Moreover, while women tend to respond to stress with higher anxiety and depressiveness, men are more reluctant to report emotional distress. In our study population only 15 % showed more than 10 points, which meant that the majority were non-depressive subjects, and 95 % were male. This uneven sampling may have underpowered our analysis.

This study has several limitations. First of all, this study evaluated CAC change over two years of follow up but as atherosclerotic calcification of vessels may require a long-standing pathological process, two year of observation might be too short to find any change in CAC. This cohort consisted of relatively healthy individuals, these results cannot be easily applied to other populations. In addition, in the study there may be a selection bias from having healthy workers. We defined CAC progression as a CAC change of > 0, and this includes one-point changes. However, a one-point increase in CAC score is not clinically meaningful and the extent of CAC increases differ according to the baseline CAC score [30]. A subtype analysis may help to elucidate a more clear association. Also as data for food intake was via a self-administered questionnaire, it is not free of recall bias and measurement error. However, this type of bias would be non-differential, biasing the estimate towards the null. Finally, we did not have information on the intake of macronutrients subtypes, as the types of fatty acids (saturated vs. unsaturated) and types of carbohydrate (complex vs. simple sugars) may differently affect preclinical atherosclerosis.

## Conclusions

In summary, the presented data showed that levels of macronutrient intake, physical activity, depressive symptoms were not associated with progression of CAC. Despite several limitations, this study is strengthened by having a large cohort of relatively healthy Korean adults. To our knowledge, this is the first study that investigates associations between macronutrient intake and progression of CAC in Koreans. Further research is needed to confirm our findings.

## Additional file

Additional file 1:
**Multivariate hazard ratios (HRs) for CAC score according to quartile of macronutrients intake (two year follow up data).** (DOCX 19 kb)

## Abbreviations

ALT: alanine aminotransferase
BMI: body mass index
CAC: coronary artery calcium
CES-D: center for epidemiology studies-depression
CVD: coronary vascular disease
DBP: diastolic blood pressure
FFQ: food frequency questionnaire
GTP: gamma glutamyl transferase
HDL-C: high-density lipoprotein cholesterol
hsCRP: high sensitivity C-reactive protein.
IPAQ: International Physical Activity Questionnaire
LDL-C: low-density lipoprotein cholesterol
MDCT: multidetector computed tomography
METS: metabolic equivalent scores
SBP: systolic blood pressure
TG: triglyceride
